# Cytokinin Type-B Response Regulators Promote Bulbil Initiation in *Lilium lancifolium*

**DOI:** 10.3390/ijms22073320

**Published:** 2021-03-24

**Authors:** Guoren He, Panpan Yang, Yuwei Cao, Yuchao Tang, Ling Wang, Meng Song, Jing Wang, Leifeng Xu, Jun Ming

**Affiliations:** 1 Institute of Vegetables and Flowers, Chinese Academy of Agricultural Sciences, Beijing 100081, China; hgr0222@sina.com (G.H.); 13067739520@163.com (Y.C.); tangyuchao100@126.com (Y.T.); 15558111707@126.com (M.S.); wangxjing126@126.com (J.W.); xuleifeng@caas.cn (L.X.); 2 School of Foresty and Landscape Architecture, Anhui Agricultural University, Hefei 230036, China; wl1985141861@126.com

**Keywords:** *Lilium lancifolium*, cytokinin, bulbil formation, type-B response regulators

## Abstract

The bulbil is an important vegetative reproductive organ in triploid *Lilium lancifolium* whose development is promoted by cytokinins. Type-B response regulators (RRs) are critical regulators that mediate primary cytokinin responses and promote cytokinin-induced gene expression. However, the function of cytokinin type-B Arabidopsis RRs (ARRs) in regulating bulbil formation is unclear. In this study, we identified five type-B *LlRR*s, *LlRR1*, *LlRR2*, *LlRR10*, *LlRR11* and *LlRR12*, in *L. lancifolium* for the first time. The five *LlRR*s encode proteins of 715, 675, 573, 582 and 647 amino acids. All of the regulators belong to the B-I subfamily, whose members typically contain a conserved CheY-homologous receiver (REC) domain and an Myb DNA-binding (MYB) domain at the N-terminus. As transcription factors, all five type-B LlRRs localize at the nucleus and are widely expressed in plant tissues, especially during axillary meristem (AM) formation. Functional analysis showed that type-B *LlRR*s are involved in bulbil formation in a functionally redundant manner and can activate *LlRR9* expression. In summary, our study elucidates the process by which cytokinins regulate bulbil initiation in *L. lancifolium* through type-B LlRRs and lays a foundation for research on the molecular mechanism of bulbil formation in the lily.

## 1. Introduction

*Lilium lancifolium* is well known for its beautiful flowers, edible bulbs and medicinal uses [1,2]. Triploid *L. lancifolium* is completely sterile and difficult to breed sexually; however, it can be propagated via asexual reproduction because it produces a large number of bulbils [3]. In *L. lancifolium*, bulbils originate on leaf axils and can fall off the ground, leading to the reproduction of new plants when bulbils mature, and each mother plant can produce dozens to hundreds of bulbils [4,5,6]. Therefore, bulbils are an important structure for breeding in *L. lancifolium*. Bulbils are only formed in a few plant species, such as *Dioscorea batatas*, *Allium sativum*, *Titanotrichum oldhamii*, *Pinellia ternate*, *Agave tequilana* and *Lilium* species [6,7,8,9,10]. Only a few studies have been conducted on the formation of bulbils to date, which have focused on the morphology of bulbils during development and exogenous hormone treatments, and only a small number of related genes have been reported [6,7,9,10,11].

The bulbil is a special, important reproductive organ in *L. lancifolium* that grows on the leaf axil as an axillary organ and originates from the axillary meristem (AM) [11,12]. Some studies have shown that auxins and cytokinins are involved in bulbil formation and suggested that auxin inhibits bulbil formation [13,14], whereas cytokinin stimulates the formation of bulbils [12,15,16]. In *T. oldhamii*, after apical dominance is broken, bulbils will quickly form from the floral primordium [13]. In *A. tequilana*, the cutting of pedicel tissue leads to the formation of bulbils at bracteoles, while the application of exogenous auxin to cut pedicel tissue suppresses bulbil formation [14]. In *L. lancifolium*, our previous work revealed that exogenous 6-benzylaminopurine (6-BA) promotes the formation of bulbils and that the cytokinin synthesis inhibitor lovastatin can inhibit this process, which is consistent with the findings of other studies in *Dioscorea zingiberensis* and *Solanum tuberosum* [12,15,16].

Cytokinins are a class of plant hormones that are involved in many aspects of plant growth and development, such as shoot and root growth, vascular tissue development, initiation of the AM, leaf development, light signal transduction, circadian rhythms, plant defense, abiotic stress and nutrient absorption [12,17,18,19,20,21,22,23,24]. The cytokinin signal transduction pathway occurs through a multistep phosphorelay similar to the two-component signaling systems (TCSs) of prokaryotes [25]. In *Arabidopsis thaliana*, the multistep phosphorelay involves three components in plants: Arabidopsis histidine kinases (AHKs), Arabidopsis histidine phosphotransfer proteins (AHPs) and Arabidopsis response regulators (ARRs) [19]. Cytokinin binds to the cyclase/histidine kinase-associated sensory extracellular (CHASE) domain of HKs and then activates the His residue of the transmitter domain, resulting in its autophosphorylation. A phosphate is subsequently transferred to the Asp residue of the HK receptor domain. HPs then receive the phosphate and transfer it to an Asp residue of an RR [26,27]. ARRs are divided into three groups: type-A, type-B and type-C [28]. Type-A ARRs, transcriptionally induced by cytokinins, are the primary cytokinin response genes and negatively regulate cytokinin signaling to reduce sensitivity to cytokinins [29,30,31,32]. Type-C ARRs are similar to type-A ARRs, but their expression is not induced by cytokinins [28,33]. Type-B ARRs are positive regulatory transcription factors in cytokinin signaling that can directly bind to target DNA sequences to activate target gene expression, including that of type-A ARRs [25,34].

Type-B ARRs are characterized by the presence of a phosphate receiver domain (CheY-homologous receiver (REC) domain) and a long C-terminal extension that contains an Myb-like DNA-binding domain (MYB) [34,35]. Phylogenetic analysis reveals an orthologous relationship among type-B ARRs in *A. thaliana* and *Oryza sativa*, and type-B ARRs are divided into five subfamilies: B-I (ARR1, ARR2, ARR10, ARR11, ARR12, ARR14 and ARR18; OsRR21, OsRR22, OsRR23, OsRR24, OsRR25 and OsRR26), B-II (ARR13 and ARR21), B-III (ARR19 and ARR20), B-V (OsRR31, OsRR32 and OsRR33) and B-IV (OsRR28 and OsRR29) [30,36,37,38]. Studies have shown that the members of subfamily B-I, especially ARR1, ARR2, ARR10, ARR11 and ARR12, are involved in cytokinin signaling as positive regulators in plants [39,40,41,42,43,44,45]. Type-B ARRs regulate a variety of plant developmental processes in a functionally redundant manner; these processes include root elongation [36,43], lateral root formation [42,43], hypocotyl elongation [45,46], shoot apical meristem development [25,43], in vitro shoot regeneration [47] and axillary shoot meristem formation [24], among others. A recent study in *A. thaliana* revealed that type-B ARRs are key regulators that participate in the initiation of the AM [24]. In the *arr1-4* mutant, a lack of *ARR1* expression leads to the failure to express *WUSCHEL* and initiate the AM. Further research showed that type-B ARRs (ARR1, ARR2, ARR10, ARR11 and ARR12) can directly bind *WUS* and activate its expression to activate the stem cell niche and complete AM initiation [24].

Our previous study showed that cytokinins can promote the initiation of the AM during the formation of bulbils in *L*. *lancifolium* [12]. Combined with the observation of positive cytokinin regulation through type-B ARRs, we speculate that exogenous cytokinins regulate bulbil initiation through type-B ARRs. Based on our transcriptome data, we cloned five type-B *LlRR*s (*LlRR1*, *LlRR2*, *LlRR10*, *LlRR11* and *LlRR12*) via RNA ligase-mediated rapid amplification of cDNA ends (RLM-RACE) and performed phylogenetic analysis, subcellular localization experiments and expression pattern analysis. Furthermore, the roles of five type-B *LlRR*s in bulbil formation were studied with a virus-induced gene silencing (VIGS) system. In addition, we found that a large number of type-B ARRs bind elements in a type-A *LlRR* (*LlRR9*) promoter and that type-B LlRRs can activate *LlRR9* expression. This is the first study to reveal a regulatory cytokinin feedback mechanism involved in the establishment of the AM during bulbil formation in the genus *Lilium*. Our study lays a foundation for further research on the molecular mechanism of bulbil formation in the lily.

## 2. Results

### 2.1. Full-Length Cloning and Sequence Analysis of Type-B ARRs

**Figure 1:**
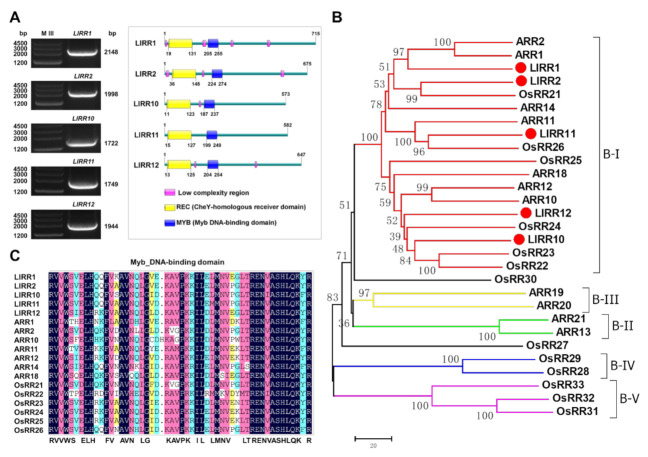
Full-length cloning and bioinformatic analyses of type-B LlRRs. (**A**) Full-length amplification and domain analyses. (**B**) Neighbor-joining tree of type-B Arabidopsis response regulator (ARR) amino acid sequences of *Lilium* *lancifolium*, *Arabidopsis thaliana* and *Oryza sativa*. (**C**) Multiple sequence alignment of the MYB domain in the B-I subfamily. Sequences from *L. lancifolium* are LlARRs that are designated with a red triangle; sequences from *A. thaliana* are shown with ARRs; sequences from *O. sativa* are shown with OsRRs. Bootstrap values from 1000 replicates were used to assess the robustness of the tree.

**Figure 2:**
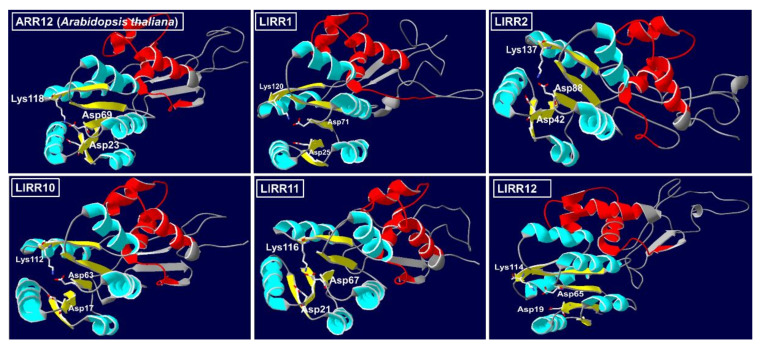
Predicted 3-D structure of ARR12 and five type-B LlRR proteins. The colors used in the predicted 3-D structure models indicate the different structures. Yellow indicates the five N-terminal β-sheets in the CheY-homologous receiver (REC) domain, and conserved Asp, Asp and Lys residues are located at the C-terminal ends of three β-sheets; blue indicates the five N-terminal α-helixes in the CheY-homologous receiver (REC) domain; red indicates the C-terminal Myb DNA-binding (MYB) domain.

We cloned the full-length sequences of *LlRR1* (2148 bp), *LlRR2* (2028 bp), *LlRR10* (1722 bp), *LlRR11* (1749 bp) and *LlRR12* (1944 bp) through RLM-RACE (Figure 1A) (GenBank: MW509629; MW509630; MW509631; MW509632; MW509633). These five *LlRR*s encode proteins of 715, 675, 573, 582 and 647 amino acids, respectively. The peptide analysis showed that LlRR1, LlRR2, LlRR10, LlRR11 and LlRR12 all contain two typical domains: an REC domain at the N-terminus and an MYB domain at the C-terminus (Figure 1A), consistent with the sequence alignments between *LlRR1*, *LlRR2*, *LlRR10*, *LlRR11* and *LlRR12* and the type-B *ARR* family members of *A. thaliana* (Supplemental Figure S1). We further performed phylogenetic analysis among the five type-B LlRRs and the type-B ARR family members of *A. thaliana* and *O. sativa*. The results show that LlRR1, LlRR2, LlRR10, LlRR11 and LlRR12 all belong to subfamily B–I (Figure 1B). Furthermore, we carried out sequence alignments of the MYB domain with the five type-B LlRRs and all members of subfamily B-I against *A. thaliana* and *O. sativa* sequences. Our results reveal that MYB domains were highly conserved in dicots and monocots (Figure 1C). Furthermore, the 3-D structure models of ARR12 and the five type-B LlRR homologs were predicted. The results indicate that the structures of the five type-B LlRRs were very similar to those of ARR12. In the N-terminal REC domain, the five type-B LlRRs and ARR12 mostly adopted a structure containing five β-sheets (yellow in Figure 2) surrounded by five α-helixes (blue in Figure 2), and the conserved residues Asp, Asp and Lys were located at the C-terminal ends of three β-sheets. There were three α-helixes in the C-terminal MYB domain (red in Figure 2), which were responsible for nuclear localization and binding to their target DNAs.

### 2.2. Subcellular Localization of Type-B ARRs

**Figure 3:**
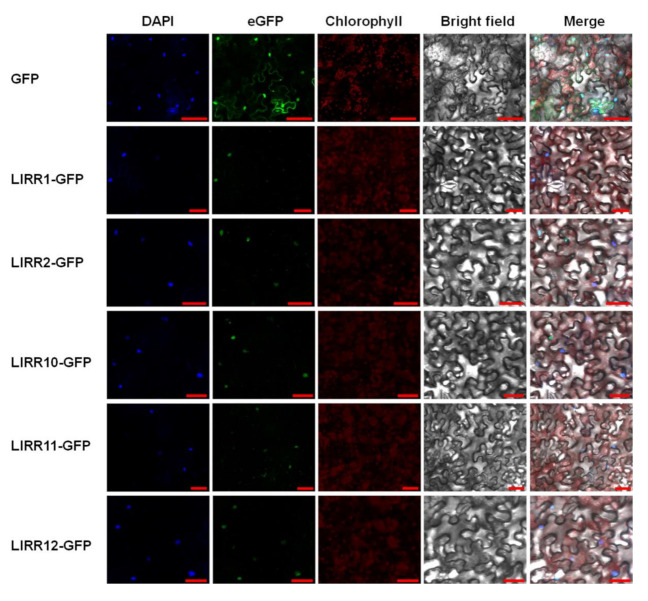
Localization of five type-B LlRR–GFP proteins in *Nicotiana benthamiana* leaf epidermal cells. The localization of the nuclei was detected by 4′,6-diamidino-2-phenylindole (DAPI) staining. DAPI: blue fluorescence signal. Blue fluorescence indicates the location of the nucleus stained by DAPI; GFP: GFP fluorescence signal. Green fluorescence indicates the location of GFP in the *N. benthamiana* leaf epidermal cells; Chlorophyll: chlorophyll autofluorescence signal. Red fluorescent signal indicates the location of chloroplasts in leaf epidermal cells. Scale bar, 50 μm.

To examine the subcellular localization of five type-B LlRRs, we fused five LlRR proteins (LlRR1, LlRR2, LlRR10, LlRR11 and LlRR12) with a green fluorescent protein (GFP) tag and introduced the construct into *N. benthamiana* leaves. The subcellular localization results show that the GFP signals of the five type-B LlRR–GFP fusion proteins all preferentially localized to the nucleus in *N. benthamiana* epidermal cells (Figure 3), confirming that these type-B LlRR proteins were located in the nucleus. These results suggest that all of the five type-B LlRRs may function in the nucleus as transcription factors.

### 2.3. Expression Pattern of Five LlRRs

**Figure 4:**
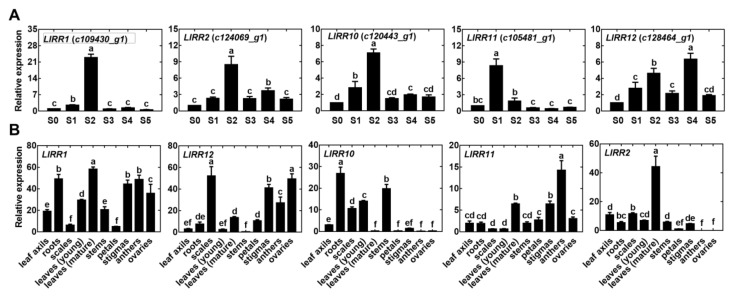
Expression patterns of five type-B *LlRR*s in *L. lancifolium*. (**A**) The expression of five type-B *LlRR*s during bulbil formation. The stages of bulbil formation were divided into the bulbil initiation stage (S0–S2), the bulbil primordium formation stage (S3–S4) and the bulbil structure formation stage (S5). (**B**) The expression of five type-B *LlRR*s in different tissues. Values are means ± SDs (*n* = 3). Lowercase letters (a–d in **A**; a–f in **B**) indicate statistically significant differences at *p* < 0.05.

The expression of the five type-B *LlRR*s was studied during bulbil formation and in different tissues. The results show that the expression of the five type-B *LlRR*s increased in the stage of meristem initiation (S0–S2) but decreased in the stage of meristem formation and bulbil scale differentiation (except *LlRR2* and *LlRR12*) (Figure 4A). It is suggested that all five type-B *LlRR*s are mainly involved in the initiation of the AM during bulbil formation in *L. lancifolium*. The tissue-related expression patterns of the five type-B *LlRR*s indicated that all five type-B *LlRR*s were expressed in all the examined tissues (Figure 4B), among which *LlRR1* and *LlRR12* were expressed in almost all the tested tissues. *LlRR2* and *LlRR10* were mainly expressed in vegetative organs and were detected at low levels in reproductive organs; however, *LlRR11* was mainly expressed in reproductive organs. Except for *LlRR10*, the expression of other type-B *LlRR*s in mature leaves was significantly higher than that in young leaves.

### 2.4. Type-B LlRRs as Positive Regulators of Bulbil Formation

**Figure 5:**
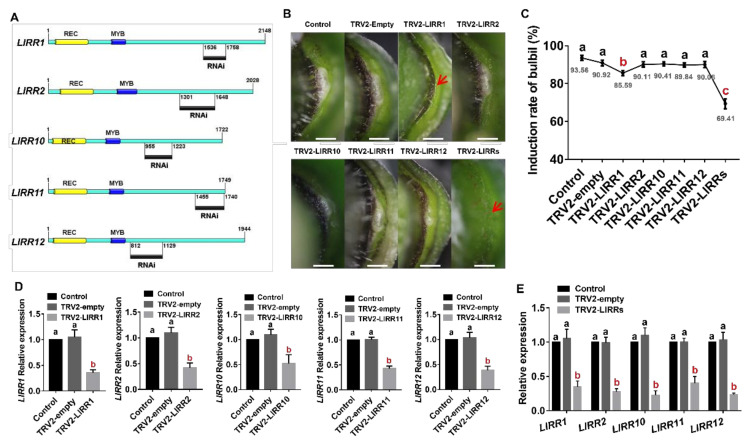
Phenotype and expression analyses after VIGS treatment. (**A**) The gene-specific fragments used in the VIGS experiment. (**B**) The phenotype of leaf axils after VIGS treatment. (**C**) The rate of bulbil formation after two weeks of culture. (**D**) Gene expression after the silencing single type-B *LlRR*s. (**E**) Gene expression after the silencing of the five type-B *LlRR*s. Values are means ± SDs (*n* = 3). Scale bar in B, 1 mm. The red arrow in B indicates that no bulbil formation is observed on the leaf axil. Lowercase letters (a–c in **C**; a–b in **D**,**E**) indicate statistically significant differences at *p* < 0.05.

To study whether the five type-B *LlRR*s are involved in the formation of bulbils, we carried out VIGS experiments. We designed specific primers in the nonconservative regions of *LlRR1*, *LlRR2*, *LlRR10*, *LlRR11* and *LlRR12* to construct the TRV2-*LlRR1*, TRV2-*LlRR2*, TRV2-*LlRR10*, TRV2-*LlRR11* and TRV2-*LlRR12* silencing vectors (Figure 5A). Our results show that after silencing either single type-B *LlRRs* alone or the five type-B *LlRR*s together, the expression of the corresponding silenced genes was decreased (Figure 5D,E). The silencing of single type-B *LlRRs* had no effect on the formation of bulbils (except for the silencing of *LlRR1*), and the induction rate of bulbils was not different from that in the control treatment (Figure 5B,C). However, after the silencing of type-B *LlRR*s, the rate of bulbil induction decreased significantly (Figure 5B,C). In addition, we found that the rate of bulbil induction decreased significantly after silencing *LlRR1* (Figure 5C). These results indicate that the function of type-B *LlRR*s in bulbil formation is redundant and that *LlRR1* may play a key role in this process.

### 2.5. A Cytokinin Feedback Loop Involved in the Initiation of the AM

**Figure 6:**
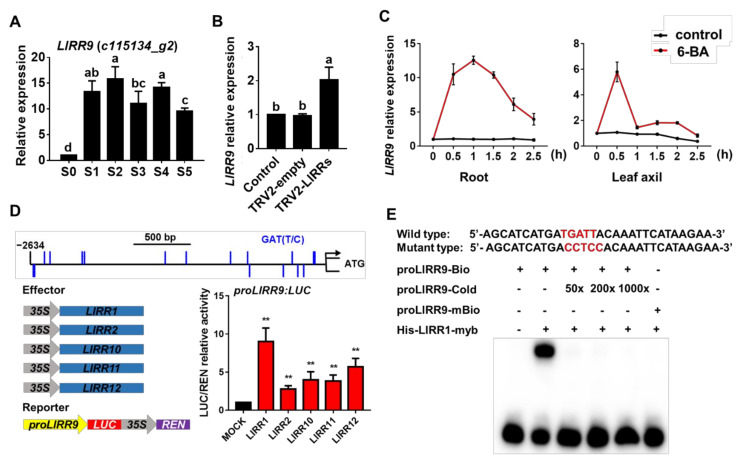
Type–B LlRRs activate the expression of *LlRR9*. (**A**) Expression of *LlRR9* during bulbil formation. S0–S2: bulbil initiation stage, S3–S4: bulbil primordium formation stage, S5: bulbil structure formation stage. (**B**) Expression of *LlRR9* after type-B LlRR silencing. (**C**) The expression of *LlRR9* was rapidly induced in both root and leaf axils after 5 mM 6-BA treatment. (**D**) Transient activation assays in *Nicotiana benthamiana* leaves. The *LlRR9* promoter contains a large number of type-B ARR binding elements (GAT(T/C)), and the five type-B LlRRs significantly enhanced *proLlRR9:LUC* activity in the transient expression system. (**E**) EMSA of LlRR1-His with the *LlRR9* promoter region. EMSA confirmed that LlRR1 directly bound to the *LlRR9* promoter region (679 to 708 bp upstream of the ATG start codon). Lowercase letters (a–c in **A**; a–b in **B**) indicate statistically significant differences at *p* < 0.05. Asterisks in **D** indicate significant differences compared with the control, with two asterisks indicating *p* < 0.01.

Type-A ARRs are transcriptionally induced by cytokinins and are the primary response genes of type-B ARRs. To study whether type-A *LlRR*s are involved in the regulation of bulbil formation, we detected the expression of type-A *LlRR*s during the process of bulbil formation in the transcriptome data and found that the expression of *LlRR9* increased during bulbil formation (Figure 6A). The increased expression of *LlRR9* after the silencing of the type-B *LlRR*s indicates that *LlRR9* may be the downstream target gene of type-B LlRRs during bulbil formation (Figure 6B), and the expression of *LlRR9* could be rapidly induced by 6-BA in roots and leaf axils (Figure 6C). When New PLACE and PlantCARE were used to analyze the *LlRR9* promoter, we found that the *LlRR9* promoter contained a large number of type-B ARR binding elements (Figure 6D) (GenBank: MW509634). Then, we carried out transient activation assays in *N. benthamiana* leaves and an EMSA assay. The results show that all five type-B LlRRs could significantly promote the activity of *LlRR9* (Figure 6D), and EMSA confirmed that LlRR1 could directly bind to the promoter of *LlRR9* (Figure 6E).

## 3. Discussion

As positive transcription factors involved in cytokinin signaling, type-B ARRs mediate the main cytokinin-induced response. However, the research on the function of type-B *ARR*s has mainly been conducted in the model plants *A. thaliana* and *O. sativa*, and less of this work has involved non-model plants [48,49]. As an important and special vegetative reproductive organ in plants, there has long been a lack of research on the regulatory mechanism on the formation of the bulbil. In this study, our results show that type-B LlRRs positively regulated bulbil initiation via partial functional redundancy and revealed a cytokinin feedback regulatory loop involved in the process of bulbil formation.

Type-B ARRs are divided into five subfamilies in *A. thaliana* and *O. sativa*, where only members of the B-I subfamily show high homology, while the other subfamilies present poor homology, indicating that type-B ARRs have undergone lineage-specific expansion in dicots and monocots [37,38]. In our study, we cloned five type-B *LlRR*s in *L. lancifolium* by RLM-RACE (Figure 1A), and phylogenetic analysis showed that they belonged to the B-I subfamily (Figure 1B). Among these LlRRs, only LlRR1 was closely related to *A. thaliana*, and LlRR2, LlRR10, LlRR11 and LlRR2 were more closely related to *O. sativa*, which supported the lineage-specific expansion of type-B ARRs in dicots and monocots.

Previous genetic studies have shown that the B-I subfamily mediates the main cytokinin signal in *A. thaliana* and *O. sativa* and that the mutant phenotypes of *arr1-3* and *arr12-1* mutants in *A. thaliana* can be restored by the overexpression of the rice B-I subfamily gene *OsRR22*. Combined with the high homology of the B-I subfamily, these findings indicate that members of the B-I subfamily show a conserved function in the cytokinin signal transduction network in higher plants [38,40,41,42]. The potential for the regulation of different gene sets by type-B ARRs is emphasized by the divergence within their MYB domains [34,39,48]. In our studies, we found that the five type-B response regulators present in *L. lancifolium* showed high homology with the members of the B-I subfamily in *A. thaliana* and *O. sativa* (Figure 1C), and the 3-D structure models revealed that the structures of the five type-B LlRRs were very similar to those of ARR12 (Figure 2). Thus, our results further indicate that there is a common cytokinin signal transduction mechanism between dicots and monocots.

Cytokinins are involved in a variety of plant development processes; accordingly, type-B *ARR*s are widely expressed in plant tissues and organs, and the B-I subfamily exhibits a broader expression profile than other subfamilies [36,38]. Our results show that the five type-B *LlRR*s in *L. lancifolium* also presented a broader expression profile, consistent with these previous studies [36,38], and we found that *LlRR1* and *LlRR12* were expressed in all examined tissues, indicating that LlRR1 and LlRR12 may be the most important response regulators in cytokinin signaling in *L. lancifolium*. In addition to the broader expression profile, our results show that the type-B LlRRs presented tissue-specific expression. For example, *LlRR2* and *LlRR10* were mainly expressed in vegetative tissues and were expressed at low levels in reproductive tissues; however, *LlRR11* was mainly expressed in reproductive tissues. In contrast to the high expression of type-B ARRs observed in the young developing leaves of *A. thaliana* and *O. sativa* [36,38], our results show that the expression of *LlRR1*, *LlRR2*, *LlRR11* and *LlRR12* in mature leaves was significantly higher than that in young leaves, which was consistent with the higher transcript levels of type-B ARRs found in mature thalli than in younger plants of *Marchantia polymorpha* [50].

Previous studies in *A. thaliana* have revealed that *ARR1*, *ARR2*, *ARR10*, *ARR11* and *ARR12* play important roles in a redundant manner [42,43,44,45]. In our study, we found that the expression of all five type-B *LlRR*s increased in the stage of AM initiation (S0–S2) (Figure 4A). In addition, the silencing of single type-B *LlRR*s did not decrease the rate of bulbil induction (except for the silencing of *LlRR1*). After the silencing of *LlRR1*, the rate of bulbil induction decreased significantly; however, the rate of bulbil induction was still significantly higher than that after the silencing of all five type-B *LlRR*s (Figure 5C). Taken together, these results suggest that type-B *LlRR*s may activate AM-related genes via functional redundancy to promote their initiation, in which LlRR1 may play a more critical role. This regulatory model is consistent with the initiation of the AM in *A. thaliana* [24] in which ARR1, ARR2, ARR10, ARR11 and ARR12 can bind the *WUSCHEL* (*WUS*) promoter and activate its expression, which is necessary for the initiation and integrity of the AM [24].

We noted that although the type-B *LlRR*s show functional redundancy in the initiation of the AM during bulbil formation, different members of the type-B LlRRs may be specifically activated at different periods in this process. For example, *LlRR11* was upregulated only at S1 (Figure 4A), whereas *LlRR2* and *LlRR12* were also upregulated at the stage of bulbil scale differentiation (S4, Figure 4A). Combined with the results of the silencing of *LlRR1*, we consider it possible that LlRR1 may be a more important regulator while other type-B LlRRs play redundant and specific roles in different periods during bulbil formation; this hypothesis is consistent with the viewpoint put forward by Ishida et al. [45] that type-B response regulators may not function as redundantly as we originally thought but may instead play roles at specific times and sites mediated by cytokinins. Indeed, some studies on individual type-B ARR family members in *A. thaliana* have supported this view. In *A. thaliana*, ARR1, ARR10, and ARR12 negatively and redundantly control plant responses to drought, although ARR1 plays a particularly vital role among the three response regulators [51]; ARR2 plays a specific role in the regulation of leaf senescence [40,52]; ARR12 promotes de novo shoot regeneration by binding to *WUS* and *CLAVATA3* (*CLV3*) promoters, while ARR1 competes for the binding of these promoters to inhibit this process [53,54]. Additionally, ARR1 can activate the expression of the auxin biosynthetic gene L-tryptophanamino transferase of Arabidopsis1 (TAA1), and ARR12 can combine with ARR1 to enhance this binding [55].

Most type-A ARRs are transcriptionally induced by cytokinins in plants and are the primary response genes of type-B ARRs [25,29,30,38,50,56]. Genetic analyses have indicated that most type-A ARR promoters contain multiple type-B ARR binding sites and function as negative regulators of cytokinin signaling by competing with type-B ARRs for phosphorylation by AHPs to negatively regulate cytokinin signaling [25,31,32,56,57,58,59,60]. In our study, we also found that the *LlRR9* promoter contains multiple type-B ARR binding sites (Figure 6D) and that type-B LlRRs can activate the expression of *LlRR9* (Figure 6D,E). Then, activated LlRR9 competes with type-B LlRRs for phosphorylation to negatively regulate the activity of type-B LlRRs. In addition, the evaluation of *LlRR9* expression in leaf axils during bulbil formation and the silencing of type-B LlRRs indicated that *LlRR9* participates in bulbil formation as a negative regulator.

After 6-BA treatment of roots and leaf axils (S4), *LlRR9* was significantly induced in roots and leaf axils. However, the expression of *LlRR9* in leaf axils increased rapidly and then decreased after 6-BA treatment after 0.5–1.0 h and then increased slightly again after 1.5–2.0 h. We speculate that other transcription factors may be involved in regulating the expression of *LlRR9* because in the leaf axil of the S4 stage, the leaf axillary meristem has been or has nearly been established, and type-A ARRs are negative regulating factors in the shoot meristem. In the shoot meristem regulatory network, except that type-B ARRs regulate the expression of type-A *ARR*s, WUS can inhibit the expression of *ARR15* and *ARR7*, and miR160 can activate the expression of *ARR15* [61,62].

In conclusion, five type-B *LlRR*s were identified for the first time in *Lilium*. Through phylogenetic, subcellar localization, gene expression pattern and functional analyses, the five type-B LlRRs of *L. lancifolium* were classified as important transcription factors during the formation of bulbils, acting in a functionally redundant manner. In addition, a cytokinin feedback loop was identified during bulbil formation. This study advances the understanding of the molecular mechanism by which cytokinins regulate bulbil formation in *Lilium*.

## 4. Materials and Methods

### 4.1. Plant Materials and Treatments

Bulbs of *L. lancifolium* of uniform size were harvested and buried in soil at 4 °C at the Institute of Vegetables and Flowers, Chinese Academy of Agricultural Sciences (CAAS), Beijing, China, in November 2019. Well-grown stems with a height of 10 cm were selected according to an in vitro bulbil induction system [12], and stem segments were cultured on Murashige and Skong medium for bulbil induction. The stages of bulbil formation were divided into the bulbil initiation stage (S0–S2), bulbil primordium formation stage (S3–S4) and bulbil structure formation stage (S5) [12]. Different stages of developmental bulbils and different tissues (leaf axils containing bulbils, leaves, stems, roots, scales, stigmas, ovaries, stamens and petals) were collected for RNA extraction.

To determine whether *LlRR9* is immediately induced by cytokinin, twelve-day-old roots and stem segments from the S4 stage were treated with 5 mM 6-BA or with 0.05 mM NaOH in Murashige and Skoog (MS) medium as a control. Roots and leaf axils were harvested at 0, 0.5, 1.0, 1.5 2.0 or 2.5 h.

### 4.2. Isolation of Type-B LlRRs and the LlRR9 Promoter

According to our transcriptome data (accession number: SRP103184), we designed primers by using Primer 6 to clone the full-length sequences of five type-B *LlRR*s and the promoter of *LlRR9*. The full-length sequences of the five type-B *LlRR*s were cloned via RLM-RACE using the GeneRacer^TM^ Kit (Invitrogen, Carlsbad, CA, USA) according to the kit protocol. To amplify the 5′ ends of *LlRR1* (*c109430_g1*), *LlRR2* (*c124069_g1*) and *LlRR12* (*c128464_g1*) and the 3′ ends of *LlRR1*, *LlRR2*, *LlRR10* (*c120443_g1*), *LlRR11* (*c105481_g1*) and *LlRR12*, a nested PCR program was used according to the kit protocol. To obtain the promoter sequence of *LlRR9* (*c115134_g2*), a genome walking kit (Takara, Japan) was used according to the kit protocol. To clone the promoter sequence of *LlRR9*, three gene-specific reverse primers were designed, and a nested PCR program was used according to the kit protocol. The sequences of the primers used for amplification are shown in Supplemental Table S1. The sequences were uploaded to GenBank.

The Simple Modular Architecture Research Tool (SMART, http://smart.embl.de/ (accessed on 21 February 2021)) was used for peptide analysis [63]. Phylogenetic analysis was performed using MEGA6 (http://mega6.software.informer.com/ (accessed on 21 February 2021)). Multiple sequence alignments were analyzed using the DNAMAN DNA analysis software package (DNAMAN version 6.0). The 3-D protein structure homology model was generated using the SWISS-MODEL protein structure homology-modeling server (www.swissmodel.expasy.org (accessed on 21 February 2021)). New PLACE (https://www.dna.affrc.go.jp/PLACE/?action=newplace (accessed on 21 February 2021)) [64] and PlantCARE (http://bioinformatics.psb.ugent.be/webtools/plantcare/html/ (accessed on 21 February 2021)) [65] were used to analyze the *LlRR9* promoter.

### 4.3. Real-Time RT-PCR (qRT-PCR)

Total RNA from the different tissues and leaf axils was extracted with an RNAprep Pure Plant Kit (TIANGEN, Beijing, China) according to the kit protocol, and DNA contamination was removed with RNase-free DNase I. First-strand cDNA was synthesized with a Hifair^®^Ⅱ 1st Strand cDNA Synthesis Kit (gDNA digester plus) (Yeasen, Shanghai, China) according to the kit protocol. Gene-specific primers for qRT-PCR were designed with Primer 6.0 (Supplemental Table S2). The *LilyActin* primer was used as an internal control [66], and SYBR^®^ Green Master Mix (No Rox) (Yeasen, Shanghai, China) was used in the reaction mixture according to the manufacturer’s instructions. qRT-PCR was conducted using the CFX96 Real-Time System (Bio-Rad, Hercules, CA, USA), with an initial denaturation step at 95 °C for 3 min, followed by 40 cycles of denaturation at 95 °C for 10 s, annealing at 60 °C for 20 s and extension at 72 °C for 1 min. A melting curve analysis was performed for each primer pair to confirm its specificity. The 2^−ΔΔCt^ method was used to calculate the relative expression levels of the different genes [67]. Three biological and three technical replicates were used to reduce error.

### 4.4. Subcellular Localization

The full-length cDNAs of *LlRR1*, *LlRR2*, *LlRR10*, *LlRR11* and *LlRR12*, under the control of the 35S cauliflower mosaic virus promoter, were cloned into the pCAMBIA 2300 vector using the pEASY^®^-Basic Seamless Cloning and Assembly Kit (Transgen Biotech, Beijing, China). The sequences of primer pairs used for amplification are shown in Supplemental Table S3. The resulting plasmids were transferred into *Agrobacterium tumefaciens* strain GV3101, which was then resuspended in infiltration buffer (10 mM methylester sulfonate, 10 mM MgCl_2_ and 150 mM acetosyringone, pH 5.7) at OD_600_ = 0.8 and infiltrated into *N. benthamiana* leaves. Three days after infiltration, the leaves were harvested and treated with 0.5 mg/mL DAPI (4′,6-diamidino-2-phenylindole; Sigma) [68]. A Zeiss LSM 510 confocal scanning microscope was used to collect images.

### 4.5. Virus-Induced Gene Silencing (VIGS)

For the generation of pTRV2-*LlRR1*, pTRV2-*LlRR2*, pTRV2-*LlRR10*, pTRV2-*LlRR11* and pTRV2-*LlRR12*, gene-specific fragments of ~300 bp (Figure 6A) were cloned into the pTRV2 vector using the pEASY^®^-Basic Seamless Cloning and Assembly Kit (Transgen Biotech, Beijing, China). The primer pairs used to generate the TRV vectors are shown in Supplemental Table S3. pTRV1, pTRV2 and the constructed plasmids were transferred into *A. tumefaciens* strain EHA105, which was then grown at 28 °C in YEB medium supplemented with 50 mg/L kanamycin and 50 mg/L rifampicin for 20–24 h until an OD_600_ = 1.0 was reached. *Agrobacterium* cells were collected and suspended in infiltration buffer that contained 10 mM MgCl_2_, 200 μM acetosyringone and 10 mM MES, pH 5.6. Before infection, a mixture of *Agrobacterium* cultures containing pTRV1 and pTRV2 and their derivatives at a ratio of 1:1 (*v/v*) was kept at room temperature in darkness for 4h. Stem segments of *L. lancifolium* were surface sterilized with 75% alcohol and 10% NaClO and then cut into small stem segments containing single leaf axils for vacuum infiltration. The small stem segments were submerged in an infiltration mixture containing pTRV1 and pTRV2 or their derivatives and then subjected to −50 kPa vacuum for 5 min [69]. The infiltrated segments were washed with distilled water three times for 3 min each time and were then grown on MS medium with 30 g/L sucrose and 6 g/L agar, pH 5.8, in the dark at 15 °C for 2 d, followed by growth at 22 °C under a 16/8 h light/dark cycle. The rate of bulbil formation was assessed after two weeks of culture, and RNA was extracted from leaf axils to measure the expression of the target genes. Each treatment consisted of three experimental replicates, with 30 leaf axils per replicate.

### 4.6. Dual-Luciferase Reporter Assay

The coding sequence of *LlRR1* was amplified from *L. lancifolium* cDNA and cloned into the pCAMBIA 3301 vector using the pEASY^®^-Basic Seamless Cloning and Assembly Kit (Transgen Biotech, Beijing, China). A 1463 bp fragment upstream of the start codon of *LlRR9* was introduced into the pluc-35Rluc vector using the pEASY^®^-Basic Seamless Cloning and Assembly Kit (Transgen Biotech, Beijing, China). The primers used to generate the constructs are listed in Supplemental Table S3. The constructed plasmids were transformed into *A. tumefaciens* strain GV3101. The suspension conditions were the same as those described above, and different effectors were subsequently coinfiltrated with the reporter into *N. benthamiana* leaves using a syringe. The 2-cm-diameter leaf discs were harvested and ground in liquid nitrogen 3 d after infiltration. The activities of firefly and Renilla luciferase were measured with the Dual-Luciferase Reporter Assay System (Promega) using a GloMax 20/20 luminometer (Promega).

### 4.7. Electrophoretic Mobility Shift Assay (EMSA)

To construct plasmids for the expression of the recombinant LlRR1 protein in *Escherichia coli*, the DNA fragments encoding the DNA-binding domains of LlRR1 (amino acids 190–271) [70] were amplified and cloned into the pET32a vector, which was expressed in the *Escherichia coli* strain BL21 (DE3) cell line. The primers are listed in Supplemental Table S3. Protein was induced by incubation in 1 mM isopropyl-β-d-thiogalactopyranoside (IPTG) at 16 °C at 160 rpm for 24 h. Protein purification was carried out using a Ni-NTA purification system (Qiagen) following the manufacturer’s instructions. Double-stranded oligonucleotide probes were synthesized and labeled with biotin at the 5′ end. EMSA was carried out using the LightShift^®^ Chemiluminescent EMSA Kit (Thermo Fisher Scientific, Waltham, MA, USA). Competition experiments were performed with different amounts of nonlabeled oligonucleotides. The mutated competitors shown in Figure 6E were generated by replacing five base pairs in the ARR binding elements (NGATT to CCTCC).

### 4.8. Statistical Analysis

All data are presented as the means ± standard error (SD) of at least three independent experiments. Duncan’s multiple range test at *p* < 0.05 or *p* < 0.01 was performed with the SPSS (version 17.0, USA) statistical package. *p* < 0.05 indicated significance.

## Data Availability

Publicly available datasets were analyzed in this study. The accession numbers can be found here: ARR1 (NP_850600.2, NM_180269.3); ARR2 (NP_193346.5, NM_117704.6); ARR10 (NP_194920.1, NM_119343.4); ARR11 (NP_176938.1, NM_105439.3); ARR12 (NP_180090.6, NM_128075.7); ARR13 (NP_001318296.1, NM_001336100.1); ARR14 (NP_178285.1, NM_126237.3); ARR18 (NP_200616.4, NM_125193.4); ARR19 (NP_001319180.1, NM_001333372.1); ARR20 (NP_001319821.1, NM_001340168.1); ARR21 (NP_196338.1, NM_120803.1); OSRR21 (XP_015630578.1, XM_015775092.1); OSRR22 (XP_015640894.1, XM_015785408.1); OSRR23 (XP_015625496.1, XM_015770010.1); OSRR24 (XP_015626716.1, XM_015771230.1); OSRR25 (XP_025882103.1, XM_026026318.1); OSRR26 (XP_015622017.1, XM_015766531.1); OSRR27 (XP_015639050.1, XM_015783564.1); OSRR28 (XP_015634799.1, XM_015779313.1); OSRR29 (XP_025880383.1, XM_026024598.1); OSRR30 (XP_015613931.1, XM_015758445.1); OSRR31 (XP_025875792.1, XM_026020007.1); OSRR32 (XP_015649246.1, XM_015793760.1); OSRR33 (XP_015649581.1, XM_015794095.1).

## Supplementary Materials

The following are available online at https://www.mdpi.com/1422-0067/22/7/3320/s1, Supplemental Figure S1. Sequence alignments between five type-B LlRRs and type-B *ARR* family of *A. thaliana*. Supplemental Table S1. Primers used in full-length cloning of five type-B *LlRR*s and promoter sequence cloning of *LlRR9*. Supplemental Table S2. Primers used in qRT-PCR. Supplemental Table S3. Primers used in vectors construction.
