# Supplementary material for: Cytokinin Type-B Response Regulators Promote Bulbil Initiation in Lilium lancifolium
Source: Int J Mol Sci. 2021 Mar 24;22(7):3320. doi: 10.3390/ijms22073320 (PMC8037933; doi:10.3390/ijms22073320)
Supplement: Supplementary file 1 [file ijms-22-03320-s001.zip › Revised Supplemental Figure 1.pdf]

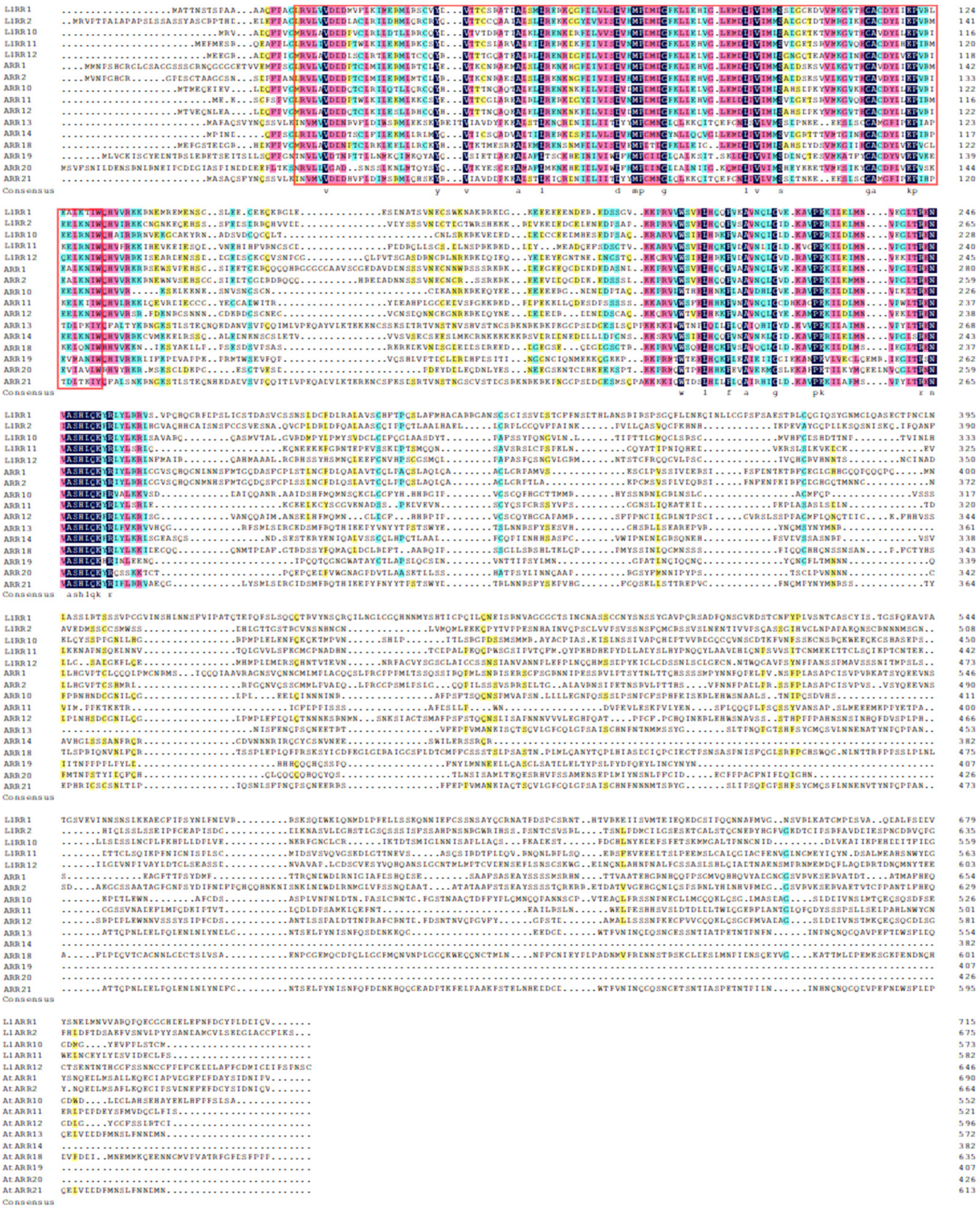

Supplemental Figure 1. Sequence alignments between five type-B *LlRR*s and type-B *ARR* family members of *A. thaliana*. The REC domain is highlighted within the red box and the MYB domain is highlighted within the grey box.
