# Supplementary material for: Cytokinin Type-B Response Regulators Promote Bulbil Initiation in Lilium lancifolium
Source: Int J Mol Sci. 2021 Mar 24;22(7):3320. doi: 10.3390/ijms22073320 (PMC8037933; doi:10.3390/ijms22073320)
Supplement: Supplementary file 1 [file ijms-22-03320-s001.zip › Revised Supplemental Tables.pdf]

Supplemental Table 1. Primers used in full length cloning of five type-B *LlRRs* and promoter sequence cloning of *LlRR9*.

Name	Sequence (5’-3’)	Purposes
5’ LlRR1-GSP1	AACCACCCTCGGCTTCTTCACC	5’RACE
5’ LlRR1-GSP2	CCTCACCACATGCTGCCAAATC	
5’ LlRR2-GSP1	ATTGCTGGAAACACTTGGCCACC	
5’ LlRR2-GSP2	TGGGCCCACTTGTGCATTTGATTCC	
5’ LlRR12-GSP1	TGGCAGTTGCATTGACCCTCCTG	
5’ LlRR12-GSP2	CTACCTCTGAGTGCCGCAGCC	
GeneRacer^{TM} 5' Primer	CGACTGGAGCACGAGGACACTGA	
GeneRacer^{TM} 5' Nested primer	GGACACTGACATGGACTGAAGGAGTA	
3’ LlRR1-GSP1	GATTTGGCAGCATGTGGTGAGG	3’RACE
3’ LlRR1-GSP2	GGTGAAGAAGCCGAGGGTGGTT	
3’ LlRR2-GSP1	GGAATCAAATGCACAAGTGGGCCCA	
3’ LlRR2-GSP2	GGTGGCCAAGTGTTTCCAGCAAT	
3’ LlRR10-GSP1	ATGAGGGTTGCAGATCAGTTTCCG	
3’ LlRR10-GSP2	CGGTGCCAGTACGATGTGACGG	
3’ LlRR11-GSP1	CTGGCGATTTTGATGACAGGCAAC	
3’ LlRR11-GSP2	GGAGGCTGATCAAGAATTCAGCGAT	
3’LlRR12-GSP1	GGCTGCGGCACTCAGAGGTAG	
3’LlRR12-GSP2	CAGGAGGGTCAATGCAACTGCCA	
GeneRacer^{TM} 3' Primer	GCTGTCAACGATACGCTACGTAACG	
GeneRacer^{TM} 3' Nested primer	CGCTACGTAACGGCATGACAGTG	
FL-LlRR1-F	ATACTCAATCCCGAATGTAATGGCC	Full length cloning
FL-LlRR1-R	CCTGAATCAGTAACACCAAAGCTG	
FL-LlRR2-F	CAGCCGCCCTATTCTTTCTCTCTC	
FL-LlRR2-R	GGGTGTTGTACAACCTAGGATTTC	
FL-LlRR10-F	ATTGGATCTTGGGGTGGATTGGTG	
FL-LlRR10-R	TCGAGCAATGCGATTGACGAACTG	
FL-LlRR11-F	TTTCTCCCCCACCATCACTTCC	
FL-LlRR11-R	AACCATTTGGAGAGAGATCACG	
FL-LlRR12-F	AGAAGATGACGGTTCCTGATAGCAG	
FL-LlRR12-R	ATTAAGCCAACTCCAAGCGACC	
proLlRR9-GSP1	CTGAGCAGCCTCTCAATGAGCTTCC	Genome walking
proLlRR9-GSP2	CTGTGACTCCGAAACAACAGCCAT	
proLlRR9-GSP3	AGCAAGATCCGACCGGATATCACGC	

Supplemental Table 2. Primers used in qRT-PCR.

Name	Sequence (5'-3')	Purposes
LIRR1-q-F	GATTTGGCAGCATGTGGTGAGG	qRT-PCR
LIRR1-q-R	CTCGCGCTCATCGTTCTCCT	
LIRR2-q-F	CAGCCCTACACTGTGCCACC	
LIRR2-q-R	AGCAGGAGCAGGATTGAGTCC	
LIRR10-q-F	TATTACCCTGTCTCGGGGCCC	
LIRR10-q-R	TGTGTCACCAGAGTTCACTTGGC	
LIRR11-q-F	AGTGCGTCGCAGAGCATCAG	
LIRR11-q-R	CTCCATGCCGTTTAGACCGAC	
LIRR12-q-F	GGCTGCGGCACTCAGAGGTA	
LIRR12-q-R	GCACACGACCATGTTGAACGAT	
LIRR9-q-F	GGGAAATCTGGAGAGCAGCCA	
LIRR9-q-R	ACAGCCAGTCTGCTAGCAGAG	

Supplemental Table 3. Primers used in vectors construction.

Name	Sequence (5'-3')	Purposes
2300-LlRR1-F	ttggagaggacagggtacccggg ATGGCCACCACCAACTCTACCT	Subcellular localization
2300-LlRR1-R	ccatggtactagtgtcgactctaga CTACACTTGAATGTCATCCAAGGGA	
2300-LlRR2-F	ttggagaggacagggtacccggg ATGAGGGTGCCGACGCC	
2300-LlRR2-R	ccatggtactagtgtcgactctaga GGATTTCAGGAAACATCCAGCTAAC	
2300-LlRR10-F	ttggagaggacagggtacccggg ATGAGGGTTGCAGATCAGTTTCCG	
2300-LlRR10-R	ccatggtactagtgtcgactctaga CATGCAAGTGCTGAGAGGGAAG	
2300-LlRR11-F	ttggagaggacagggtacccggg ATGGAGTTCATGGAGAGCAGGC	
2300-LlRR11-R	ccatggtactagtgtcgactctaga CGAAAAAAGGCACTCATCAATTACC	
2300-LlRR12-F	ttggagaggacagggtacccggg ATGGAGGAAGGTCGAGCTGATC	
2300-LlRR12-R	ccatggtactagtgtcgactctaga CATGCAACTATTCGGAGAAAAGATG	
TRV2-LlRR1-F	gtgagtaaggttaccgaattc GTGAAAGACTCAACCGGGAATTTCT	VIGS
TRV2-LlRR1-R	tccccatggaggccttctaga CATGTTCTGTAACTTCCAGTCTTGG	
TRV2-LlRR2-F	gtgagtaaggttaccgaattc CCTACACTGTGCCACCAGAATCC	
TRV2-LlRR2-R	tccccatggaggccttctaga TTGATGACGATTGTGAACCGAGAG	
TRV2-LlRR10-F	gtgagtaaggttaccgaattc GGCGACAGTCATGATACAACAAACC	
TRV2-LlRR10-R	tccccatggaggccttctaga GGATCATGCTGAGGTGCAACAAT	
TRV2-LlRR11-F	gtgagtaaggttaccgaattc GTCGCAGAGCATCAGAGATACCTTT	
TRV2-LlRR11-R	tccccatggaggccttctaga AAAGGCACTCATCAATTACCGAATC	
TRV2-LlRR12-F	gtgagtaaggttaccgaattc CTGCGGCACTCAGAGGTAGACA	
TRV2-LlRR12-R	tccccatggaggccttctaga CTGTGTTGTGTTGTGATCGGTCC	
3301-LlRR1-F	cgggggactcttgaccatgg ATGGCCACCACCAACTCTACCT	Dual-luciferase reporter assay
3301-LlRR1-R	ggaaattcgagctggtcacc CACTTGAATGTCATCCAAGGGATAC	
3301-LlRR2-F	gtgagtaaggttaccgaattc CCTACACTGTGCCACCAGAATCC	
3301-LlRR2-R	tccccatggaggccttctaga TTGATGACGATTGTGAACCGAGAG	
3301-LlRR10-F	gtgagtaaggttaccgaattc GGCGACAGTCATGATACAACAAACC	
3301-LlRR10-R	tccccatggaggccttctaga GGATCATGCTGAGGTGCAACAAT	
3301-LlRR11-F	gtgagtaaggttaccgaattc GTCGCAGAGCATCAGAGATACCTTT	
3301-LlRR11-R	tccccatggaggccttctaga AAAGGCACTCATCAATTACCGAATC	
3301-LlRR12-F	gtgagtaaggttaccgaattc CTGCGGCACTCAGAGGTAGACA	
3301-LlRR12-R	tccccatggaggccttctaga CTGTGTTGTGTTGTGATCGGTCC	
Luc-proLlRR9-F	accatgattacgaattgagctc CCAGTCCAGGGGTGACTGAC	
Luc-proLlRR9-R	tttggcgtcttccatgaattc CTGTGACTCCGAAACAACAGCCAT	
pET-LlRR1myb-F	ggctgatatcggatccgaattc ATGGAGGAGAACGATGAGCGC	EMSA
pET-LlRR1myb-R	tcgagtgcggccgcaagctt ATCAAATCGTCCCTGATGTTGTGG	
